# Supplementary material for: OXA1L mutations cause mitochondrial encephalopathy and a combined oxidative phosphorylation defect
Source: EMBO Mol Med. 2018 Sep 10;10(11):e9060. doi: 10.15252/emmm.201809060 (PMC6220311; doi:10.15252/emmm.201809060)
Supplement: Supplementary file 1 — Appendix [file EMMM-10-e9060-s001.pdf]

Appendix

Table of Contents

Table S1: Transcript Summary of OXA1L Isoforms 2

Table S2: Identification of OXA1L homologues using HHPred 3

Figure S1: Bayesian phylogenetic analysis of OXA1L homologues. 4

References... 5

						Median Gene Expression (TPM-Transcripts Per Million)								
						Artery		Brain				Heart		
Name	Transcript ID	bp	Protein	Biotype	UniProt	Aorta (299)	Coronary (173)	Amygdala (100)	Cortex (158)	Spinal Cord (91)	Transformed fibroblasts (343)	Arterial Appendage (297)	Left Verticle (303)	Skeletal Muscle (564)
OXA1L-213	ENST00000612549.4	2971	495aa	Protein coding	J3KNA0	119	125.2	28.52	44.94	47.04	161.8	87.48	97.36	147
OXA1L-201	ENST00000285848.9	1719	495aa	Protein coding	J3KNA0	0	0	0	0	0	0	0	0	0
OXA1L-202	ENST00000358043.5	1750	419aa	Protein coding	Q15070-2	0.72	0.77	0.1	0.23	0.22	0.72	0.4	0.19	0.21
OXA1L-212	ENST00000604262.5	1571	435aa	Protein coding	S4R3Q9	59.23	61.62	17.04	26.35	24.59	91.73	54.97	66.09	104.5
OXA1L-203	ENST00000412791.5	1440	408aa	Protein coding	E7EVY0	0	0	0	0	0	0	0	0	0
OXA1L-204	ENST00000431881.6	889	112aa	Protein coding	C9JC63	0.35	0.36	0	0.08	0	0.26	0.15	0.08	0.26
OXA1L-205	ENST00000442110.5	691	82aa	NMD	H7BZE6	-	-	-	-	-	-	-	-	-
OXA1L-209	ENST00000495424.5	1679	No protein	Retained intron	-	0	0	0	0	0	0	0	0	0
OXA1L-207	ENST00000481218.1	1596	No protein	Retained intron	-	15.32	17.18	3.87	7.17	7.39	12.11	7.47	5.59	3.61
OXA1L-208	ENST00000483939.6	1224	No protein	Retained intron	-	3.44	3.67	0.51	1.67	1.11	5.29	1.59	1.23	0.88
OXA1L-206	ENST00000473744.5	824	No protein	Retained intron	-	0.18	0	0	0	0	0.19	0.07	0	0.23
OXA1L-210	ENST00000556473.1	593	No protein	Retained intron	-	-	-	-	-	-	-	-	-	-
OXA1L-211	ENST00000557299.5	538	No protein	Retained intron	-	1.53	1.33	0.1	0.26	0.36	1.61	0.69	0.42	0.32

Table S1: Transcript Summary of OXA1L Isoforms

Transcript Summary of OXA1L Isoforms including the Median gene Expression in transcripts per million (TPM) in selected tissues. Expression data was obtained from GTEx Portal (https://www.gtexportal.org/home/). All other data was obtained from Ensembl v.92.

	No	Species	Gene	Hit	Prob	E-value	P-value	Score
OXAL1	1	HS	OXA1L	NP_005006.3	100	7.00E-147	4.00E-152	832.8
	2	DM	CG6404, isoform A	NP_729634.1	100	7.10E-99	5.00E-104	569.4
	3	DM	CG6404, isoform B	NP_648417.1	100	7.10E-99	5.00E-104	569.4
	4	SC	OXA1	NP_011081.1	100	6.20E-80	4.20E-85	465.6
	5	HS	COX18 isoform 1	NP_001284661.1	100	8.00E-61	5.40E-66	360.8
	6	HS	COX18 isoform 2	NP_776188.1	100	1.30E-60	8.90E-66	359.6
	7	DM	CG4942	NP_648286.1	100	4.40E-59	3.00E-64	351.3
	8	HS	PREDICTED: COX18 isoformX2	XP_016863534.1	100	3.70E-53	2.50E-58	318.8
	9	HS	PREDICTED: COX18 isoformX1	XP_005265737.1	100	1.90E-53	1.30E-58	320.4
	10	SC	COX18	NP_011576.1	100	2.80E-48	1.90E-53	292
	11	HS	COX18 isoform 4	NP_001287658.1	100	7.80E-47	5.30E-52	284.1
	12	HS	COX18 isoform 3	NP_001284662.1	100	1.40E-35	9.70E-41	222.4
	13	HS	PREDICTED: COX18 isoformX5	XP_011530180.1	100	1.60E-34	1.10E-39	216.7
	14	MJ	DUF106	WP_010869981.1	97.6	0.0024	1.70E-08	45.7
DUF106	1	HS	OXA1L	NP_005006.3	99.9	3.30E-25	2.20E-30	165.6
	2	DM	CG6404, isoform B	NP_648417.1	99.9	3.80E-25	2.60E-30	165.2
	3	DM	CG6404, isoform A	NP_729634.1	99.9	3.80E-25	2.60E-30	165.2
	4	SC	OXA1	NP_011081.1	99.9	1.30E-23	8.90E-29	156.8
	5	HS	PREDICTED: COX18 isoformX2	XP_016863534.1	99.9	3.50E-22	2.40E-27	148.9
	6	HS	COX18 isoform 2	NP_776188.1	99.9	5.30E-22	3.60E-27	148
	7	HS	PREDICTED: COX18 isoformX1	XP_005265737.1	99.9	4.00E-22	2.80E-27	148.6
	8	HS	COX18 isoform 1	NP_001284661.1	99.9	8.20E-22	5.60E-27	146.9
	9	HS	TMCO1 isoform 1	NP_061899.2	99.9	1.00E-21	7.10E-27	146.4
	10	DM	Uncharacterized: Dmel_CG10470	NP_609936.1	99.9	1.50E-21	1.00E-26	145.5
	11	HS	TMCO1 isoform 2	NP_001243093.1	99.8	4.30E-21	3.00E-26	143
	12	HS	TMCO1 isoform 3	NP_001243094.1	99.8	4.50E-21	3.10E-26	142.8
	13	DM	CG4942	NP_648286.1	99.8	1.60E-20	1.10E-25	139.9
	14	SC	COX18	NP_011576.1	99.8	1.60E-18	1.10E-23	128.9
	15	HS	COX18 isoform 4	NP_001287658.1	99.4	3.50E-13	2.40E-18	99.6
	16	SC	EMC3P	NP_012715.3	99.2	1.60E-11	1.10E-16	90.5
	17	DM	EMC3P	NP_609444.1	99.2	3.60E-11	2.50E-16	88.6
	18	HS	EMC3P isoform X1	XP_005265378.1	99.1	2.60E-11	1.80E-16	89.3
	19	HS	EMC3P	NP_060917.1	99.1	3.10E-11	2.20E-16	88.9
	20	HS	COX18 isoform 3	NP_001284662.1	98.6	4.00E-08	2.70E-13	71.9
	21	HS	PREDICTED: COX18 isoformX5	XP_011530180.1	98.5	6.10E-08	4.20E-13	70.9
	22	HS	WRB isoform 1	NP_004618.2	97.6	0.002	1.40E-08	46
	23	HS	WRB isoform 2	NP_001139690.1	97.3	0.0018	1.20E-08	46.3
	24	SC	GET1	NP_011495.1	97.3	0.0091	6.20E-08	42.5

Table S2: Identification of OXA1L homologues using HHPred

HHpred analysis (Zimmermann *et al*, 2017) of . Proteomes for Human (HS), *D. melanogaster* (DM). *S. cerevisiae* (SC) and *M. Jannaschii* were searched for OXA1 (NP_005006.3) and DUF106 (WP_010869981.1) using the default 'global' alignment settings, repeating in part the analysis of Anghel *et al*., (Anghel *et al*, 2017). Hits with probability score greater than 97 were reported. The E-value, P-value and Score for each hit is also reported.

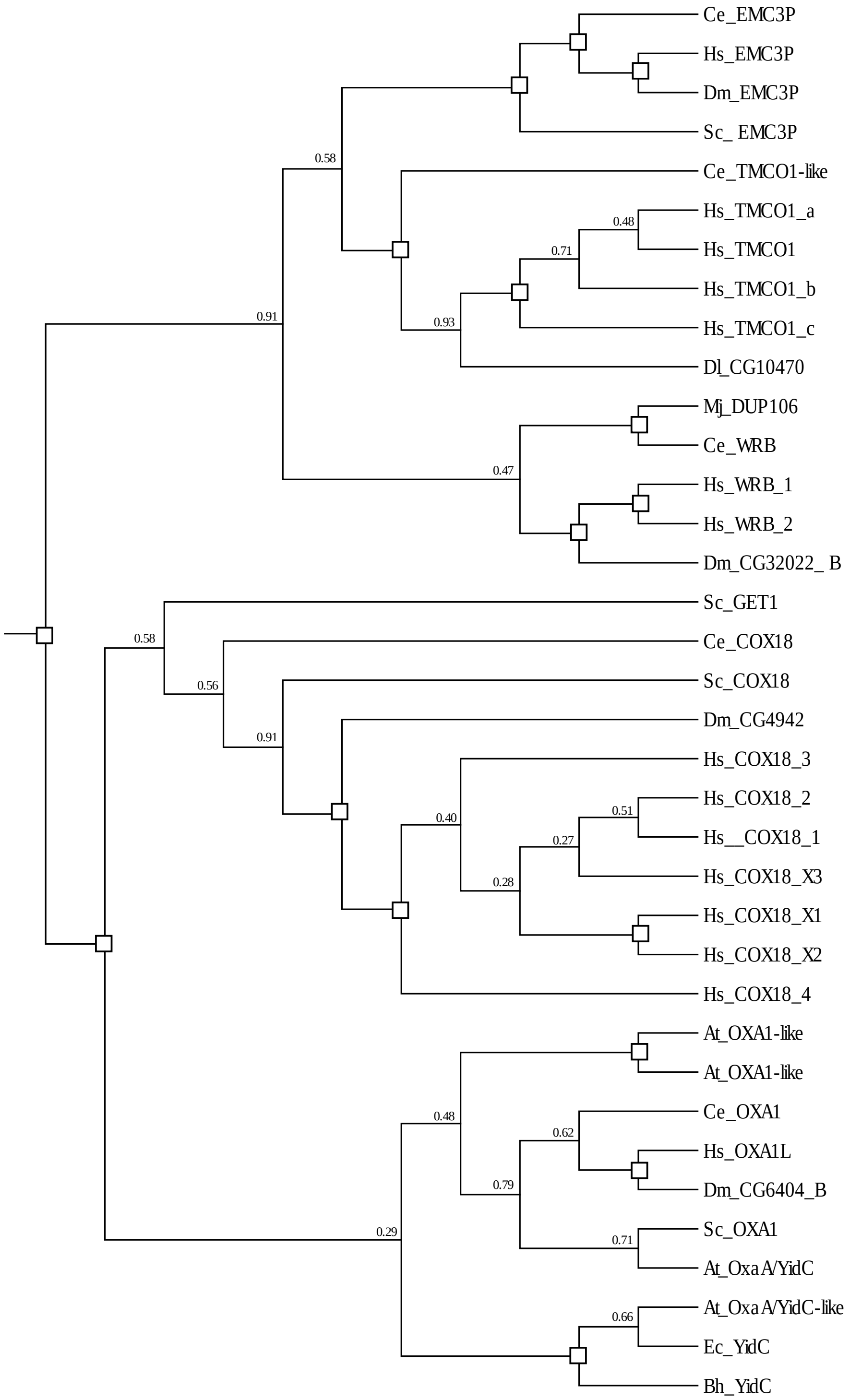

Figure S1: Bayesian phylogenetic analysis of *OXA1L* homologues.

White boxes represent posterior values >0.95. HHPred (Zimmermann *et al*, 2017) was used to identify potential OXA1L homologues in various species and protein sequences for each were obtained through the NCBI database. An alignment of 321 amino acid positions was constructed using MAFFT v.7 (Katoh & Standley, 2013), before the GUIDANCE2 algorithm (Landan & Graur, 2008; Sela *et al*, 2015) was used to identify potential regions of low confidence. All columns below 90% confidence were removed. The resulting alignment was then submitted to the IQ-Tree server which identified LG+F+G4 as the best-fitting amino acid substitution model out of the 144 models tested. BEAST (Drummond & Rambaut, 2007), was used to construct the tree with the Markov chain Monte Carlo (MCMC) chain run for 10-million generations and sampled every 1,000th generation. The species abbreviations are as follows: At = *A. thaliana*, Bh= *B. halodurans*, Ce = *C. elegans*, Dm= *D. melanogaster*, Hs = *H. sapiens*, Mj = *M. Jannaschii*, Sc = *S. cerevisiae*.

References

Anghel SA, McGilvray PT, Hegde RS & Keenan RJ (2017) Identification of Oxa1 Homologs Operating in the Eukaryotic Endoplasmic Reticulum. *Cell Rep* **21:** 3708–3716

Drummond AJ & Rambaut A (2007) BEAST: Bayesian evolutionary analysis by sampling trees. *BMC Evol. Biol.* **7:** 214

Katoh K & Standley DM (2013) MAFFT multiple sequence alignment software version 7: improvements in performance and usability. *Mol. Biol. Evol.* **30:** 772–780

Landan G & Graur D (2008) Local reliability measures from sets of co-optimal multiple sequence alignments. *Pac Symp Biocomput***:** 15–24

Sela I, Ashkenazy H, Katoh K & Pupko T (2015) GUIDANCE2: accurate detection of unreliable alignment regions accounting for the uncertainty of multiple parameters. *Nucleic Acids Res.* **43:** W7–14

Zimmermann L, Stephens A, Nam S-Z, Rau D, Kübler J, Lozajic M, Gabler F, Söding J, Lupas AN & Alva V (2017) A Completely Reimplemented MPI Bioinformatics Toolkit with a New HHpred Server at its Core. *J Mol Biol*
