# Supplementary material for: OXA1L mutations cause mitochondrial encephalopathy and a combined oxidative phosphorylation defect
Source: EMBO Mol Med. 2018 Sep 10;10(11):e9060. doi: 10.15252/emmm.201809060 (PMC6220311; doi:10.15252/emmm.201809060)
Supplement: Supplementary file 2 — Expanded View Figures PDF [file EMMM-10-e9060-s002.pdf]

Expanded View Figures

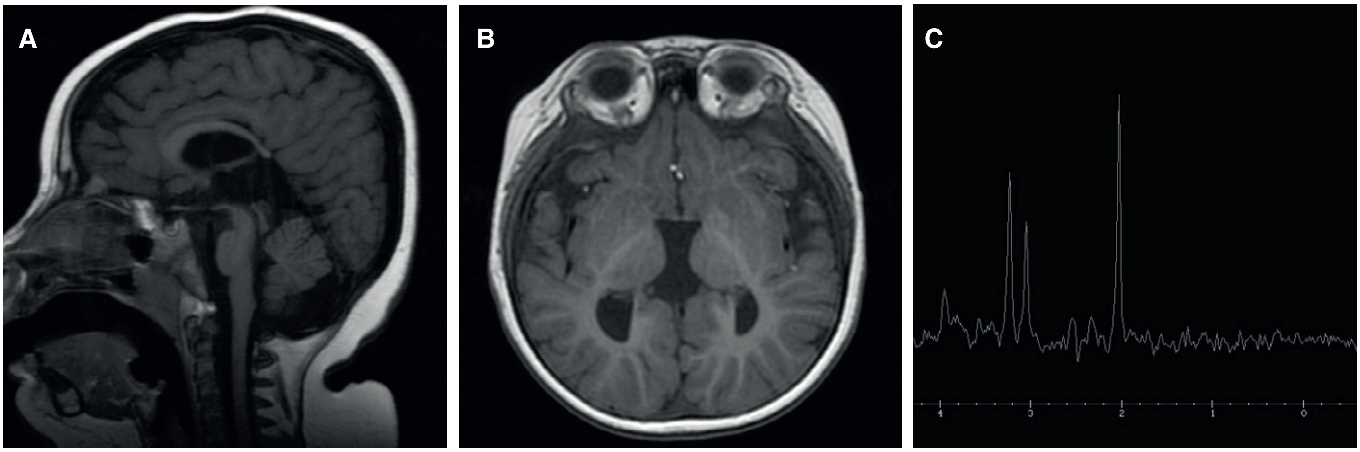

Figure EV1. Patient Neuroimaging.

A Sagittal T1-weighted FSE showing thinning of the posterior part of the corpus callosum with agenesis of the splenium. Prominent subcutaneous fat deposition can also be seen.

B Axial T1 FSPGR reveals that the third ventricle is bigger and the ventricular atrium has a parallel orientation, due to agenesis of the splenium.

C MRS of the patient's brain shows a normal spectroscopy pattern.

Figure EV2. Neuropathology: Bilateral cavitation of the pons, demyelination of the fasciculus gracile tract and downregulation of complex I subunits NDUFS3 and NDUFB8.

A The pons demonstrate bilateral cavitation (i and ii), specifically affecting the pedunculopontine nucleus, accompanied by microvascular proliferation (iii), reactive astrogliosis (iv) and microglial activation (v). There is downregulation of NDUFS3 (vi; arrowheads) expression within neurons and the surrounding neuropil (control staining: vii), while SDHA (viii; control shown in ix), COXI (x; control shown in xi) and ATP5B (xii; control shown in xiii) expression is maintained within normal levels.

B The neurons of the substantia nigra also reveal a loss of NDUFS3 (i; arrowheads, control shown on right) expression, while SDHA (ii left panel; control shown on right), COXI (iii left panel; control shown on right) and ATP5B (iv left panel; control shown on right) expression levels are comparable to control.

C The fasciculus gracile tracts reveal demyelination (i and ii), while the adjacent fasciculus cuneatus is myelinated (iii). Neuronal population density of the motor neurons is maintained, while there is a loss of NDUFB8 (iv) and NDUFS3 expression (v) and intact SDHA (vi), COXI (vii) and ATP5B (viii) expression.

Data information: Scale bar = 100 μm.

▶

© 2018 The Authors

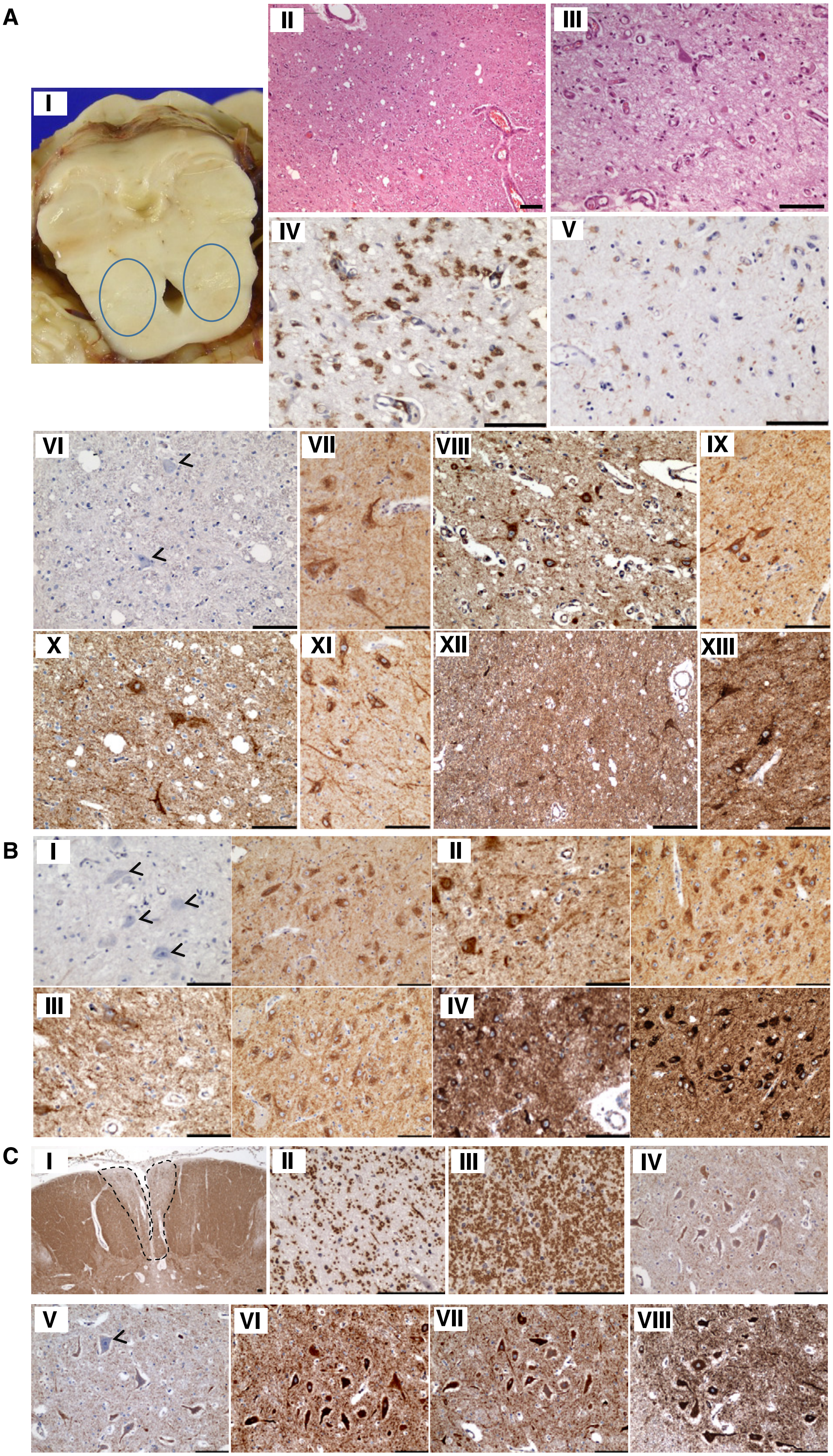
A
I
II
III
IV
V
VI
VII
VIII
IX
X
XI
XII
XIII
B
I
II
III
IV
C
I
II
III
IV
V
VI
VII
VIII

Figure EV2.

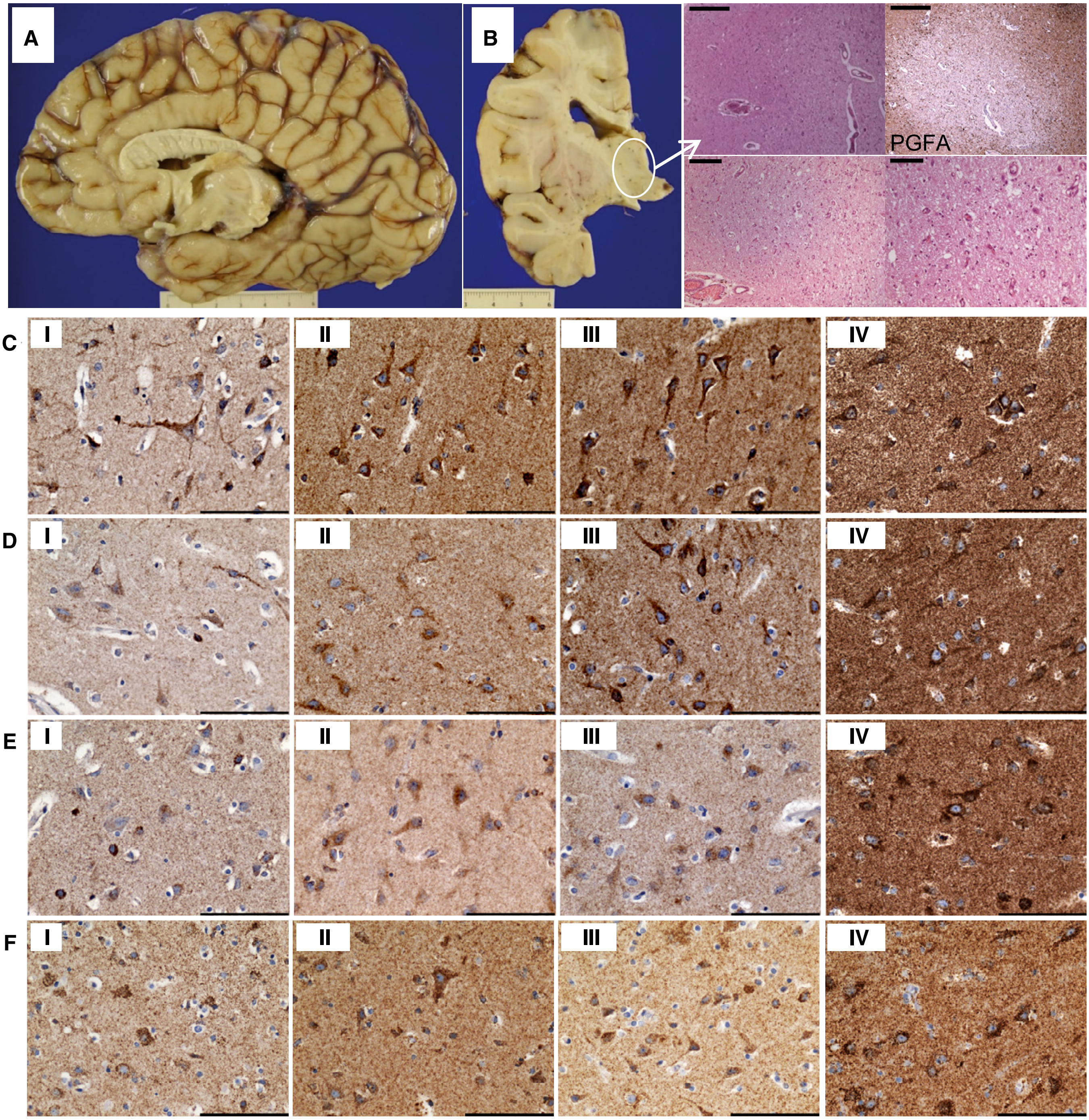

Figure EV3. Neuropathology: Frontal lobe atrophy, corpus callosum hypoplasia and thalamic cavitation. The laminar distribution of neurons in cortical regions is intact, and mitochondrial OXPHOS expression is maintained throughout the frontal, parietal and occipital cortices.

A Macroscopically, the frontal cortex was atrophic, while the corpus callosum was hypoplastic.

B There was microcavitation of the thalamus with microvascular proliferation, activated microglia and reactive gliosis, which was revealed with immunohistochemical staining for glial fibrillary acidic protein (PGFA). Scale bars: top panels = 500 μm, bottom left = 200 μm, bottom right = 100 μm.

C–F Mitochondrial respiratory chain protein expression is maintained in patient neurons within frontal (C), parietal (D) and occipital (E) cortices comparable with levels observed in control neurons (F), and there is only slight downregulation of NDUFS3 (i) expression, while expression in patient tissues of SDHA (ii), COXI (iii) and ATP5B (iv) is high. Scale bar = 100 μm.

 © 2018 The Authors

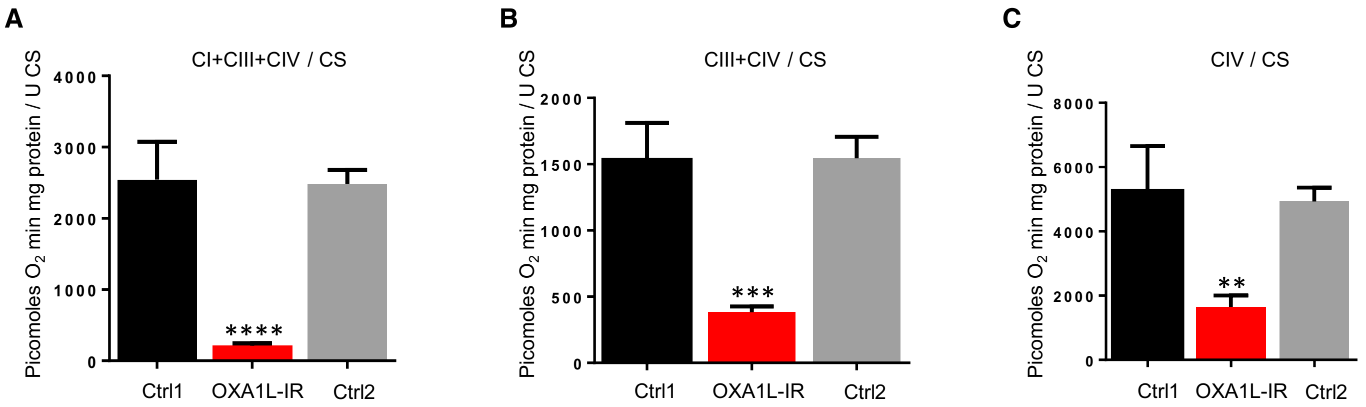

Figure EV4. High-resolution respirometry in fly homogenates of CG6404-depleted flies and controls.

A CI+CIII+CIV respiration using pyruvate+proline as substrate.
B Respiration using sn-glycerol-3-phosphate as substrate (in the presence of rotenone).
C CIV respiration using TMPD and ascorbate as substrates (in the presence of rotenone and antimycin A).

Data information: Three to five biological replicates per group. Ordinary one-way ANOVA with Tukey's multiple comparison test. Error bars represent SEM. $^{**}P < 0.01$, $^{***}P < 0.001$, $^{****}P < 0.0001$.

Figure EV5. GTEx Portal Expression Profiles for OXA1L Isoforms.
All boxplots generated using the Genotype-Tissue Expression Project (GTEx) Analysis Release V7, individual tissue eQTL dataset available through the GTEx Portal (https://www.gtexportal.org/home/).

A Boxplots of expression levels in transcripts per million (TPM) for OXA1L-213, the main protein coding isoform of OXA1L, across the full available tissue panel. Box plots are shown as median and 25th and 75th percentiles; points are displayed as outliers if they are above or below 1.5 times the interquartile range.
B–K Expression levels in TPM for isoforms OXA1L-201 through 212, respectively, across a reduced panel of tissues. The reduced tissue panel includes artery—aorta, artery—coronary, brain—amygdala, brain—cortex, brain—spinal cord, cells—transformed fibroblasts, heart—atrial appendage, heart—left ventricle and muscle—skeletal.

© 2018 The Authors

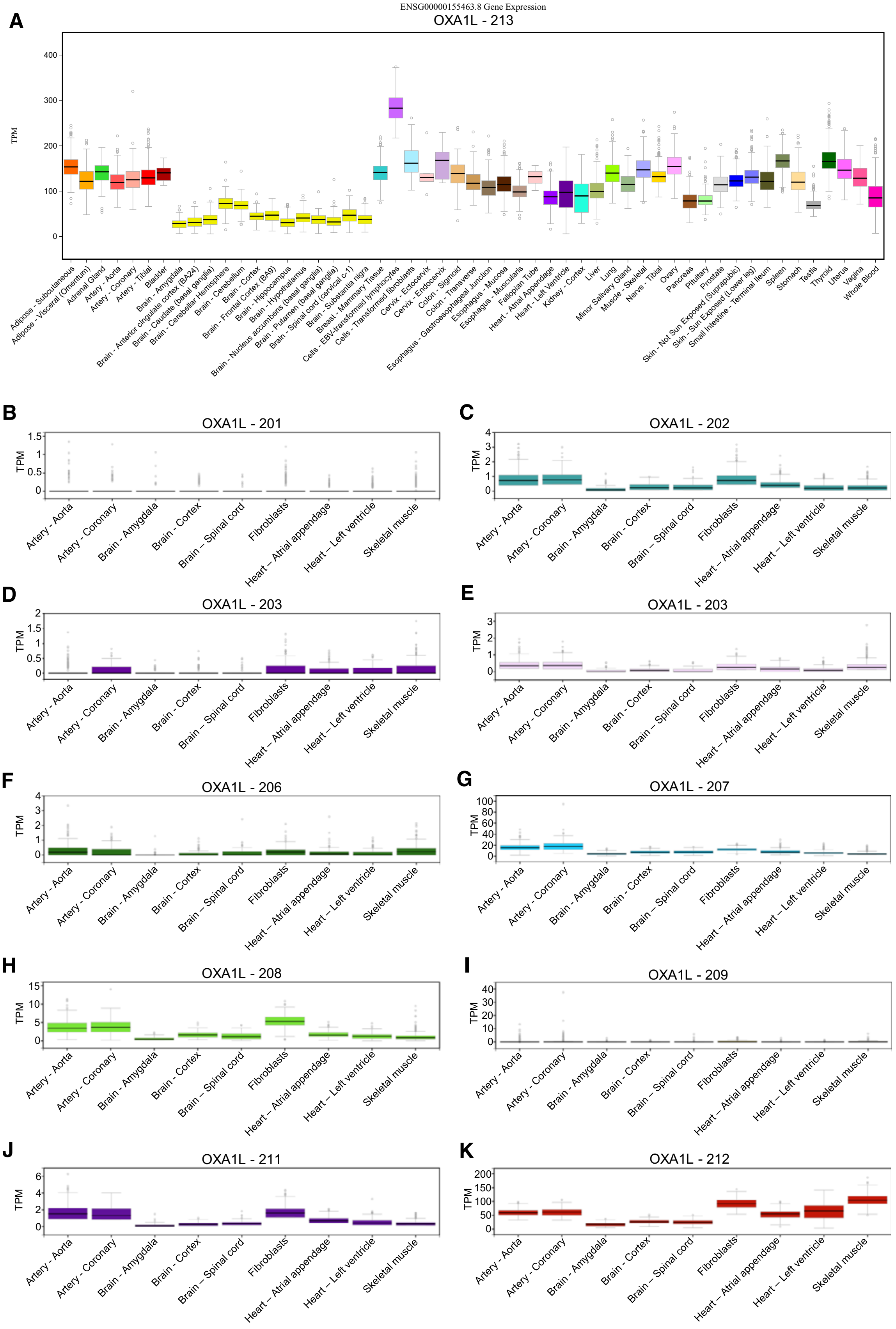

Figure EV5.

© 2018 The Authors
