# Supplementary material for: OXA1L mutations cause mitochondrial encephalopathy and a combined oxidative phosphorylation defect
Source: EMBO Mol Med. 2018 Sep 10;10(11):e9060. doi: 10.15252/emmm.201809060 (PMC6220311; doi:10.15252/emmm.201809060)
Supplement: Supplementary file 5 — Source Data for Figure 2 [file EMMM-10-e9060-s004.pdf]

Figure 2A Source Data

Chemiluminescent signal used in figure | **Colorimetric image to show MW marker** | **Merged image**

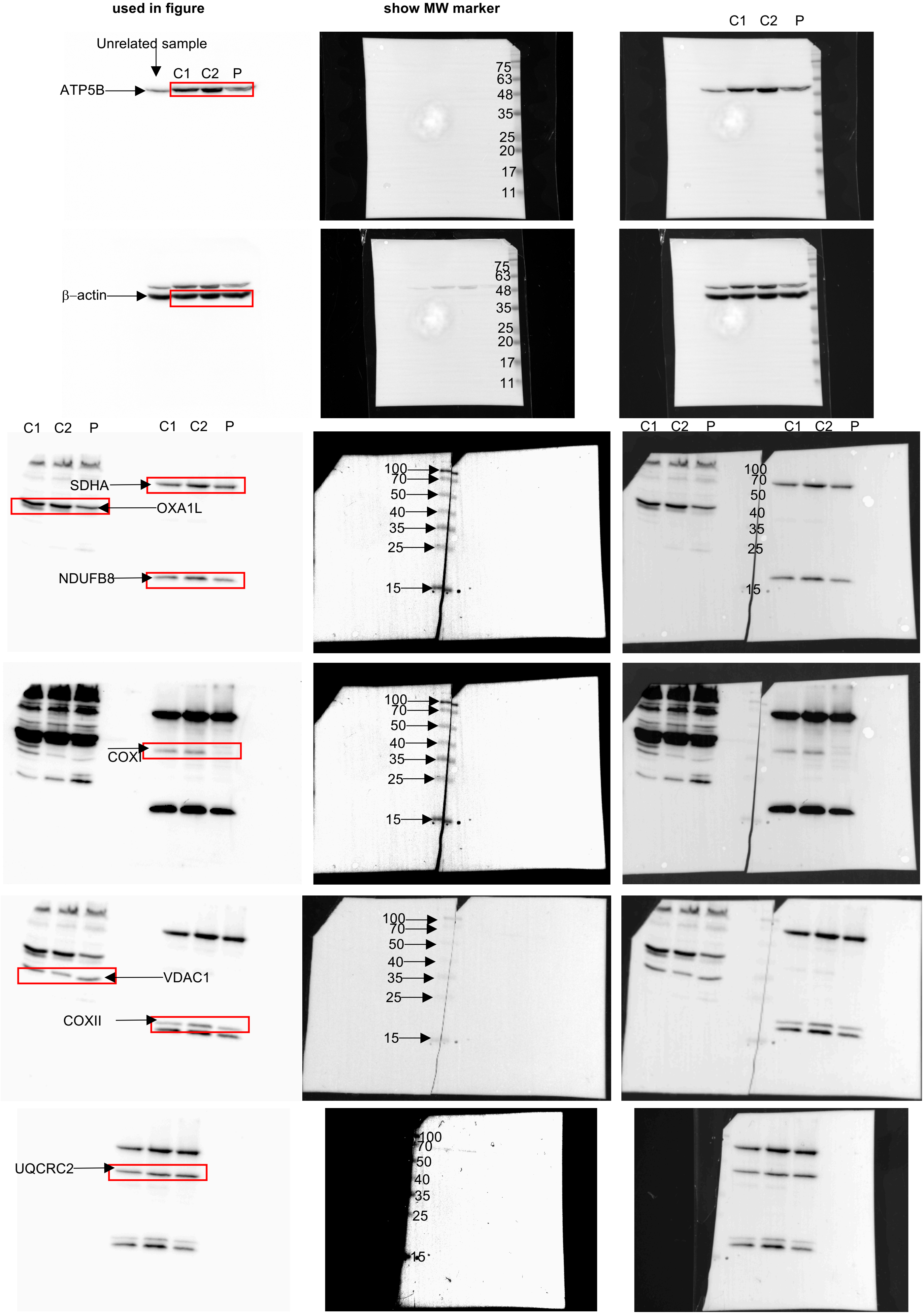

Figure 2B Source Data

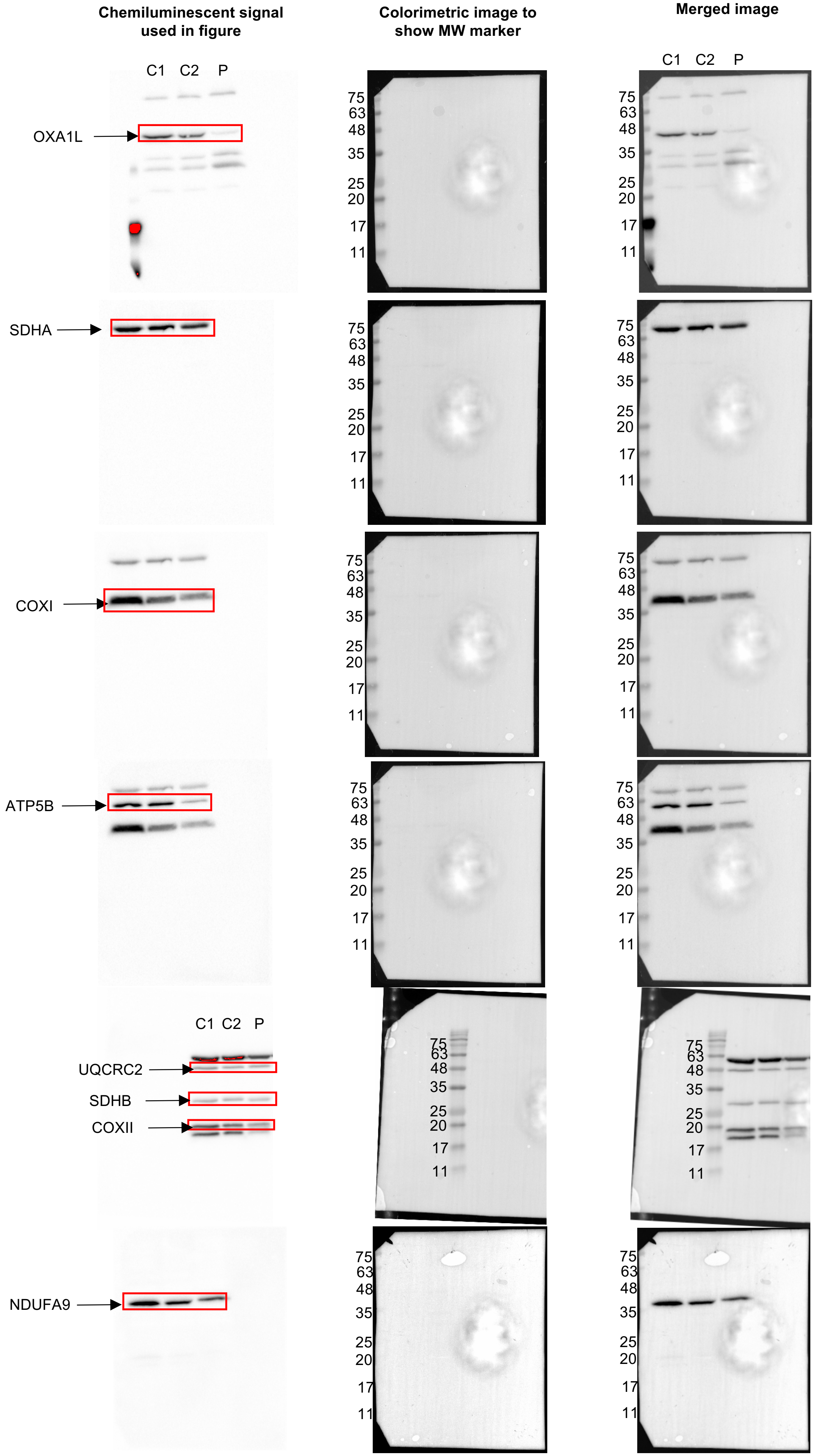

Figure 2C Source Data

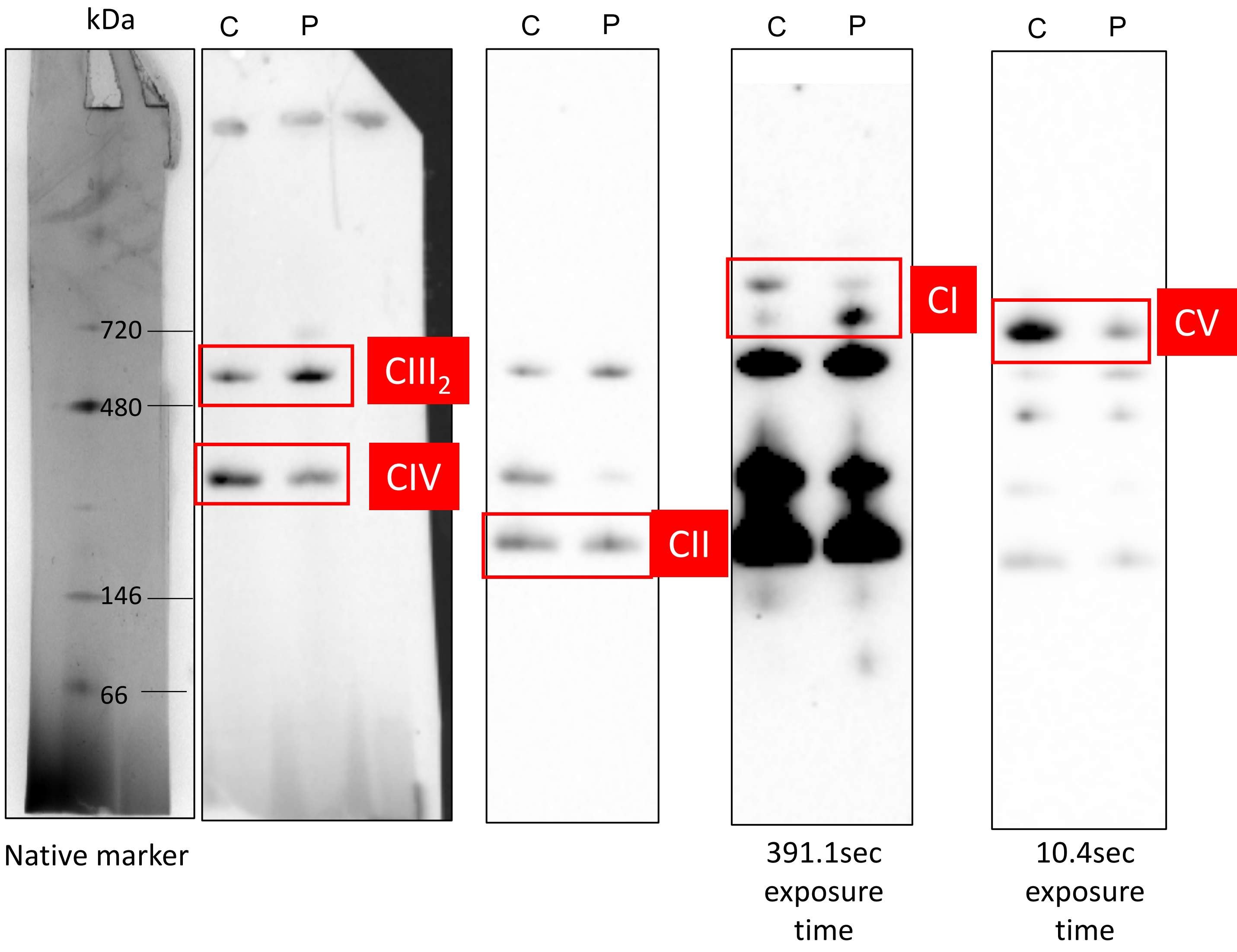

BN PAGE – control and OX1L fibroblasts
4-16% gel

CI (~980kDa) – NDUFB8
CII (~140kDa) – SDHA
CIII$_2$ (500kDa) – UQCRC2
CIV (~220kDa)– COXI
CV (~650KDa) – ATP5A

Figure 2D Source Data

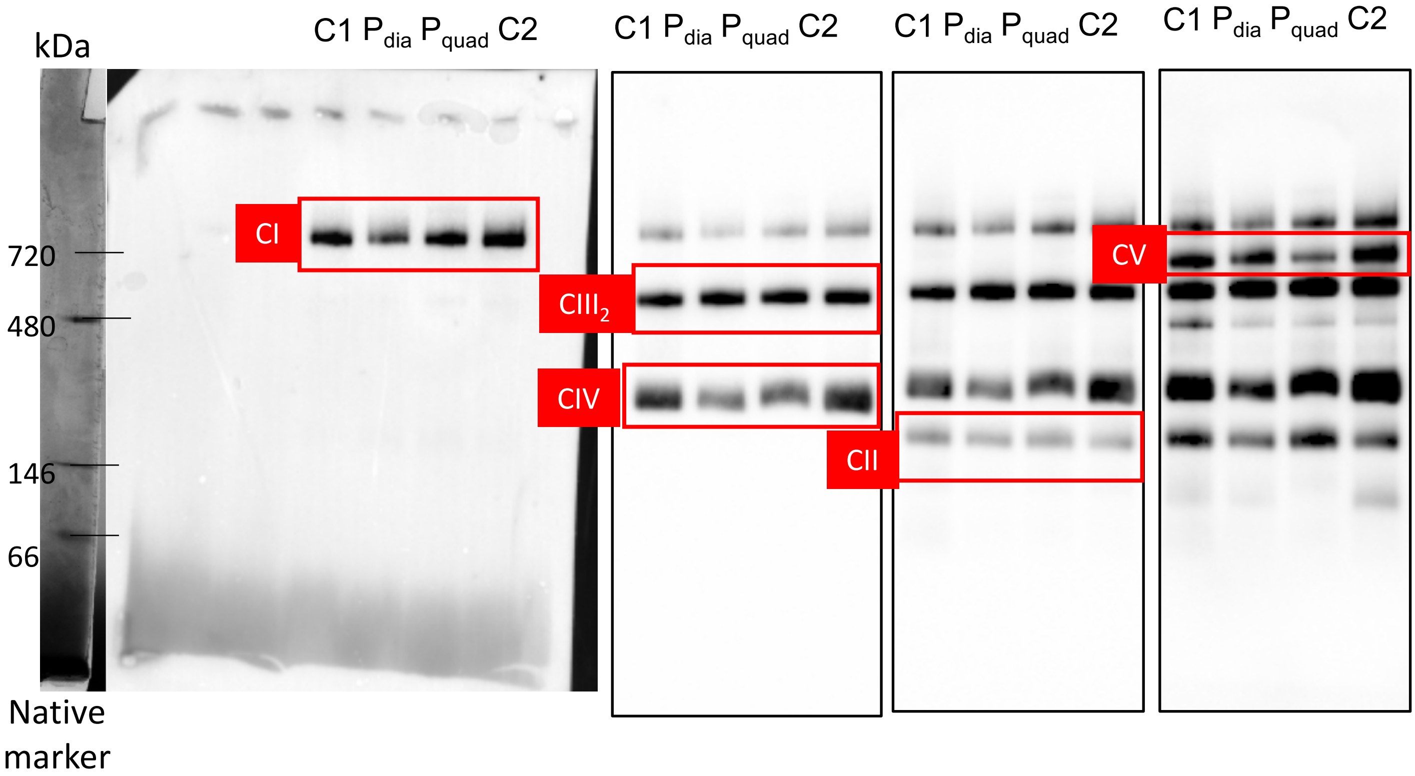

BN PAGE – Control and OXA1L muscle
4-16% gel

CI (~980kDa) – NDUFB8
CII (~140kDa) – SDHA
$CIII_2$ (500kDa) – UQCRC2
CIV (~220kDa)– COXI
CV (~650KDa) – ATP5A

Figure 2E Source Data

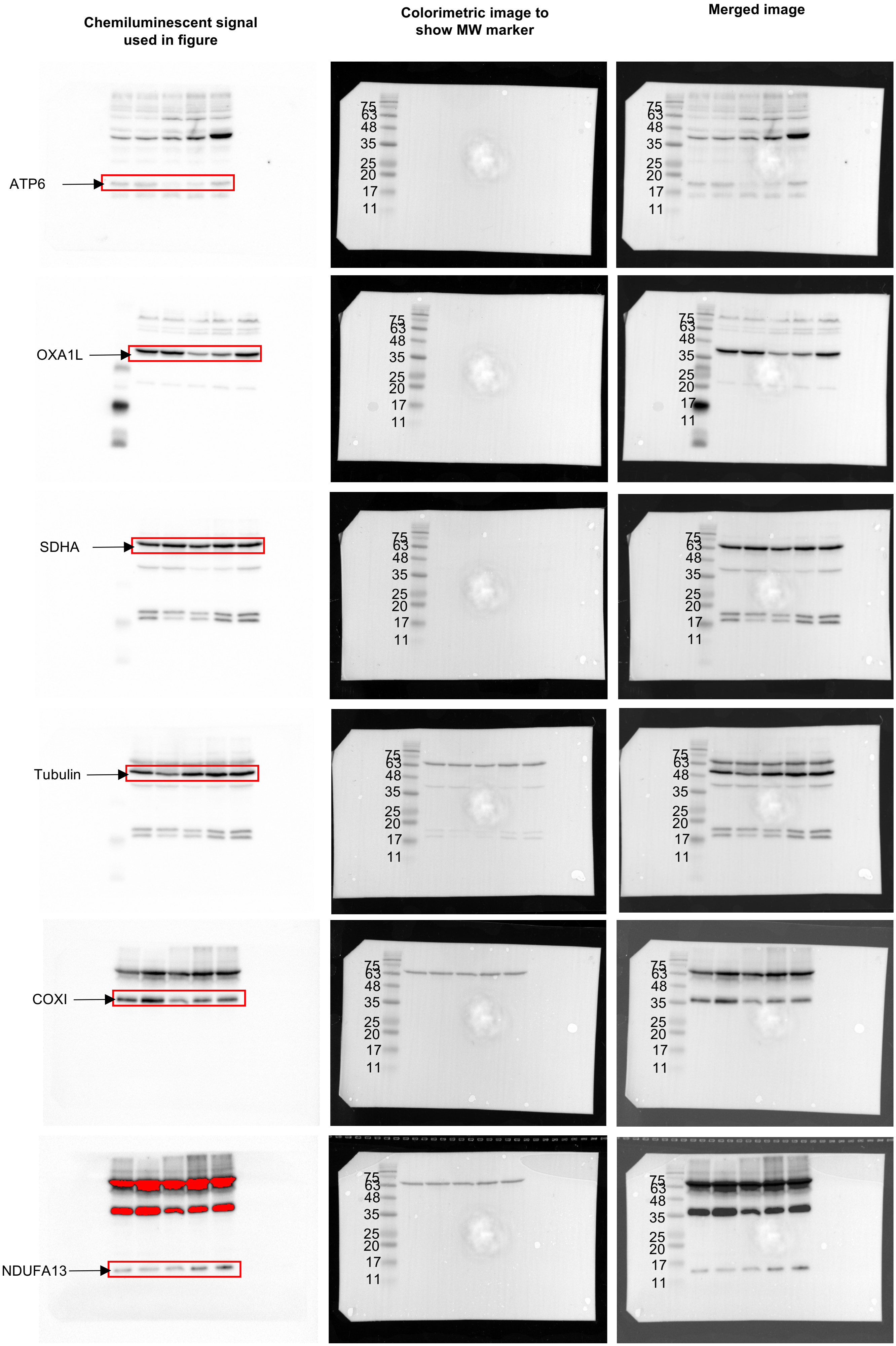

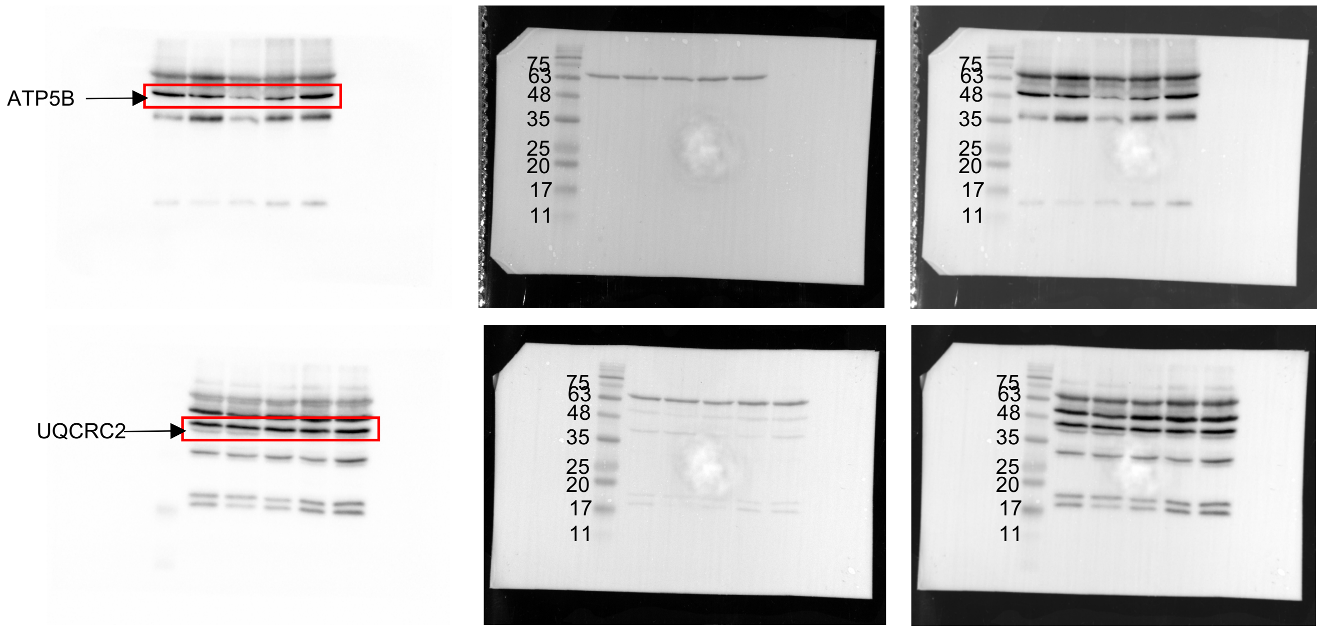
ATP5B
UQCRC2
75
63
48
35
25
20
17
11
