# Supplementary material for: OXA1L mutations cause mitochondrial encephalopathy and a combined oxidative phosphorylation defect
Source: EMBO Mol Med. 2018 Sep 10;10(11):e9060. doi: 10.15252/emmm.201809060 (PMC6220311; doi:10.15252/emmm.201809060)

Figure 3B Source Data

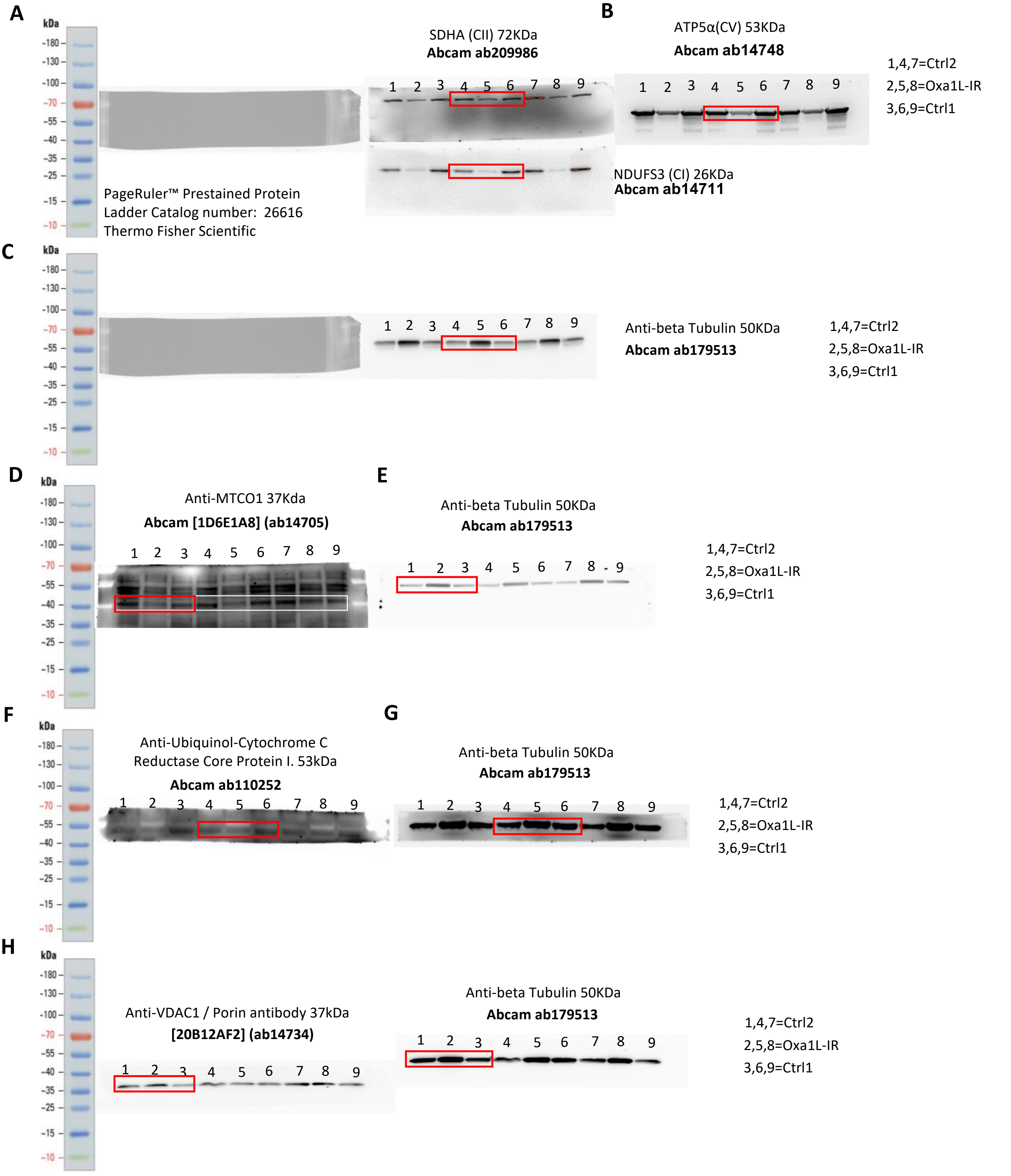

Three biological replicates per group have been used for each membrane. The first membrane has been cut and each part used for the incubation with NDUFS3 or SDHA antibody (A). The upper part of the membrane has been re-incubated with the ATP5α antibody (B) and then with Anti-beta Tubulin used as loading control (C). To increase the MTCO1 signal both the blocking and the MTCO1 incubation was done using BSA 0,5%, (by using Milk 0.5% a very faint signal was detected a the expected size). The incubation in BSA rather than milk increase the signal but gives also a dark background (D). The same membrane was then incubated with Anti-beta Tubulin in Milk 0,5% (E). To increase the signal the membrane has been cut at the expected size and both blocking and the Anti-Ubiquinol-Cytochrome C Reductase Core incubation was done using BSA 0,5% (F). The same membrane was then incubated with Anti-beta Tubulin in Milk 0,5% (G). The membrane has been cut and each part used for the incubation with Porin or Anti-beta Tubulin (H)

Figure 3C Source Data

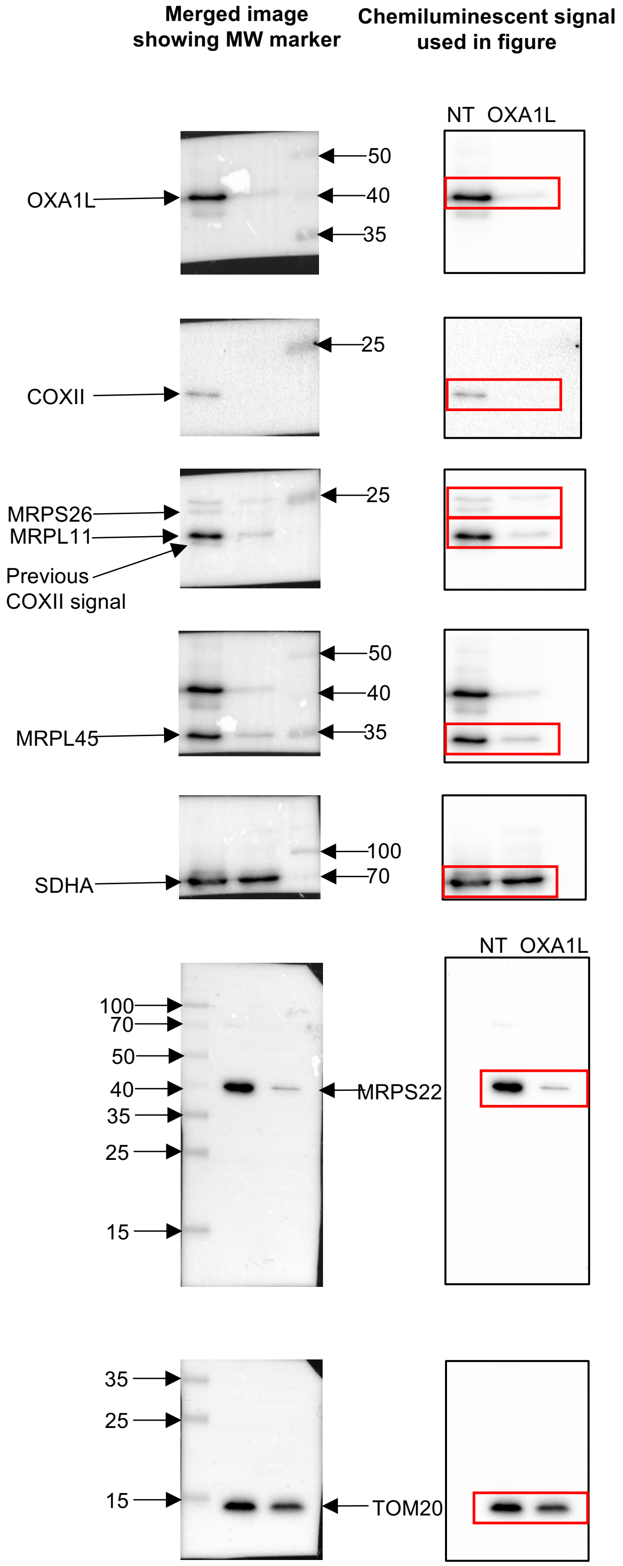

Figure 3D Source Data

Merged image showing MW marker

Chemiluminescent signal used in figure

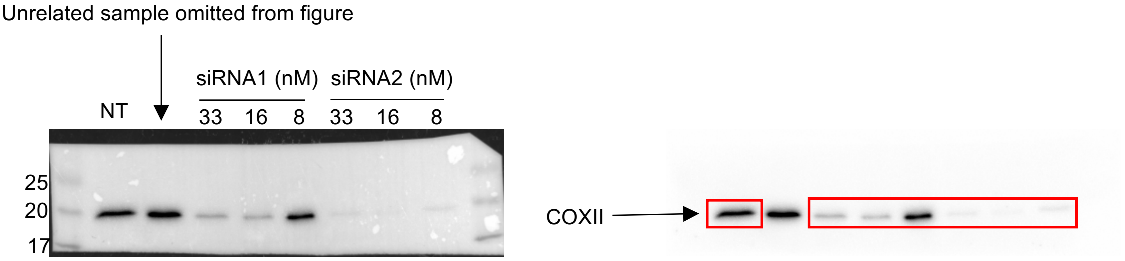

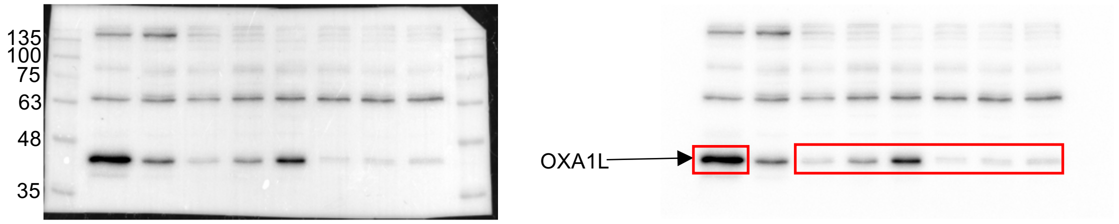

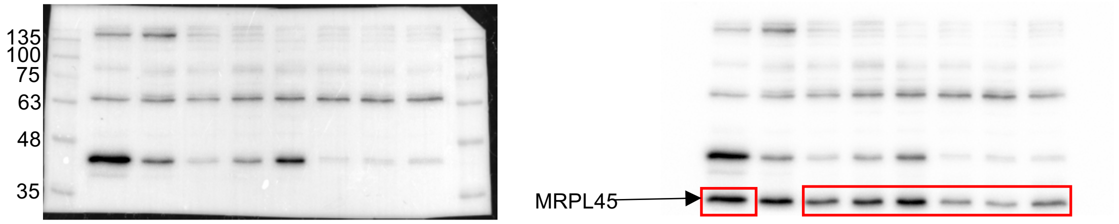

This membrane was re-probed with MRPL45 (see right panel) but the colorimetric image was not saved.

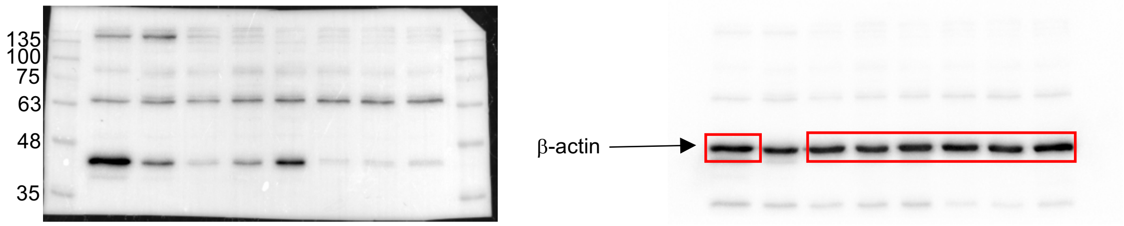

This membrane was re-probed with β–actin (see right panel) but the colorimetric image was not saved.

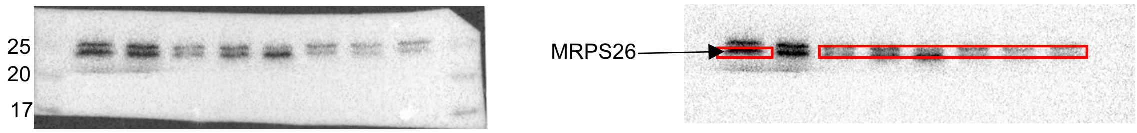

Figure 3E Source Data

Chemiluminescent signal used in figure | Colorimetric image to show molecular weight markers | Merge

Figure 3E, OXA1L blot, low exposure (10 sec)

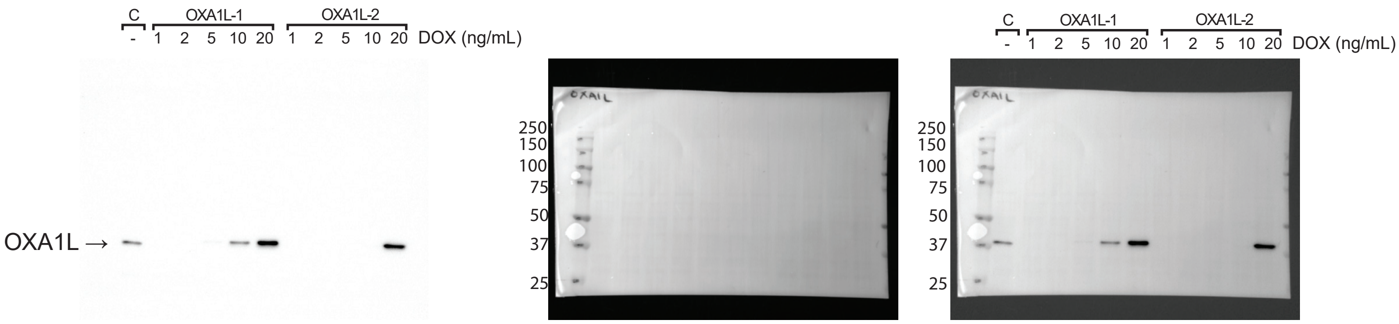

Figure 3E, OXA1L blot, high exposure (60 sec)

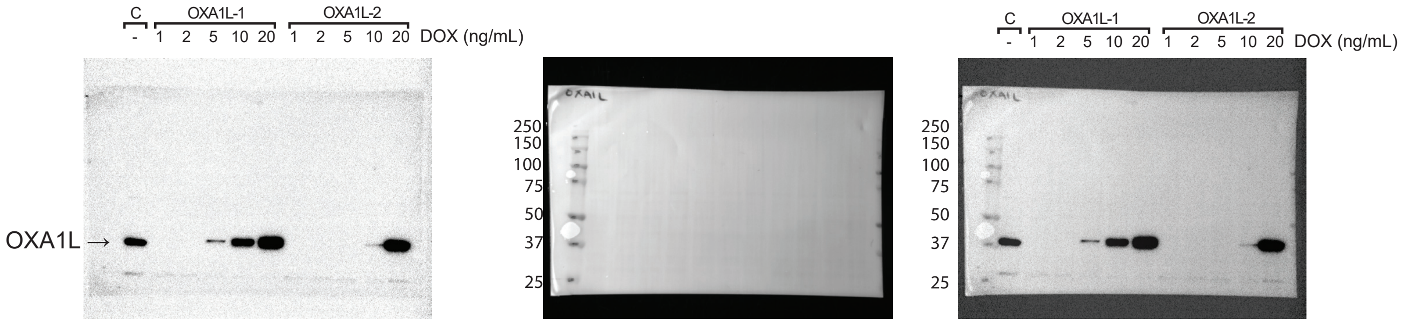

Figure 3E, TOM20 blot for both OXA1L and Flag (used as loading)

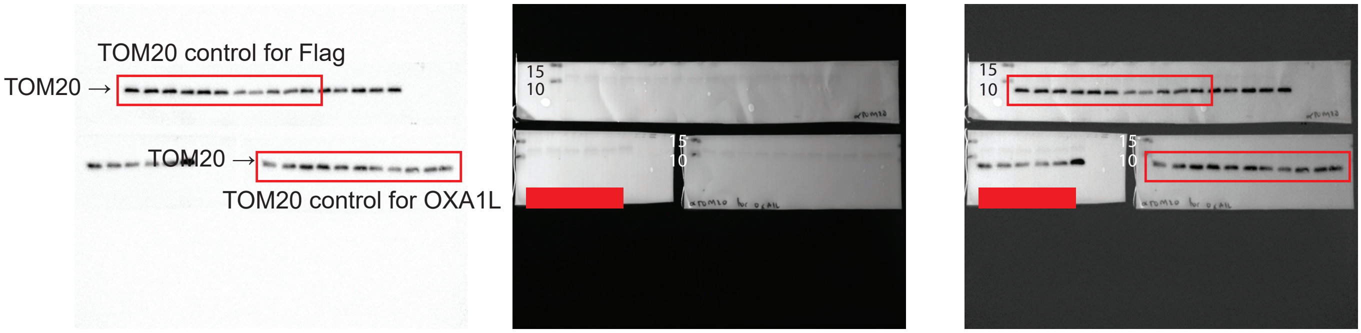

Figure 3E, Flag blot

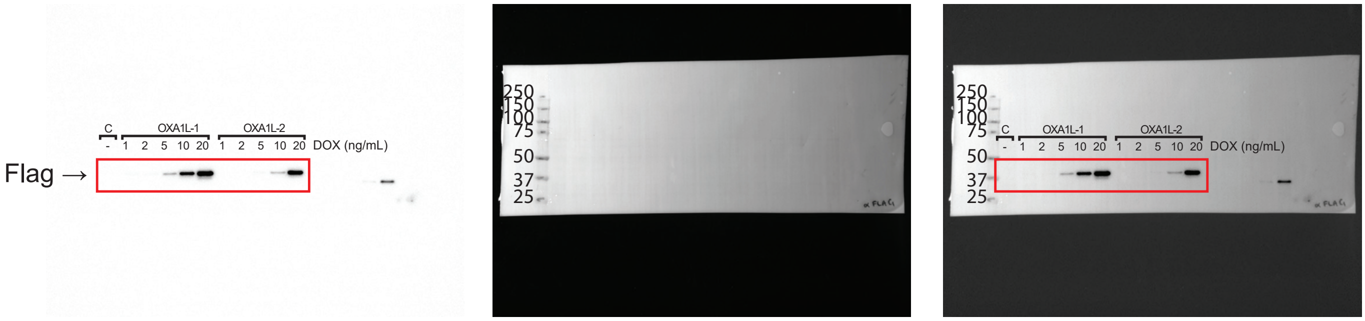

Figure 3F Source Data

Chemiluminescent signal used in figure	Colorimetric image to show molecular weight markers	Merge

Figure 3F, NDUFA9 blot

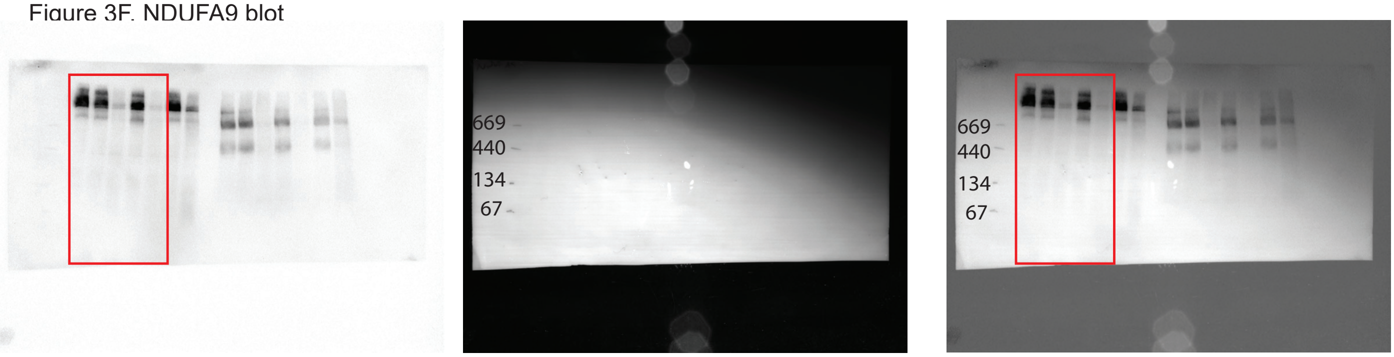

Figure 3F, UQCRC1 blot

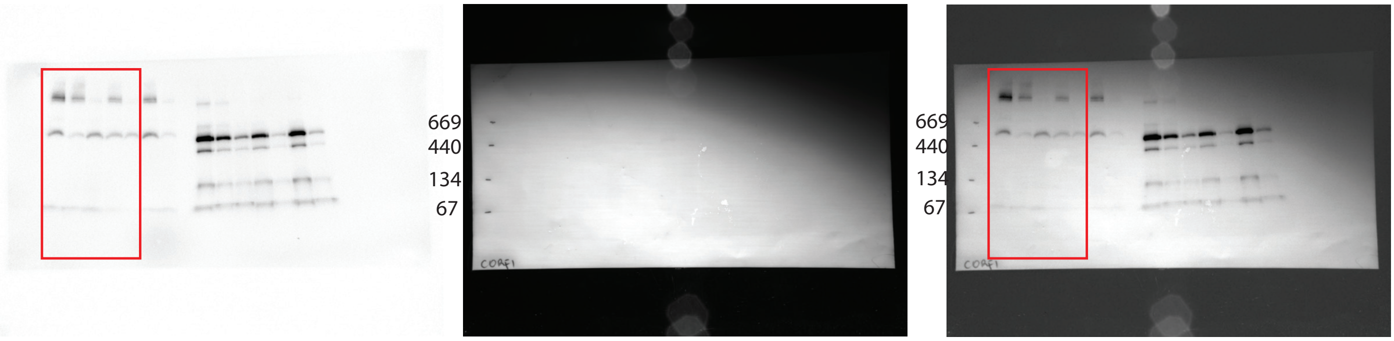

Figure 3F, COXIV blot

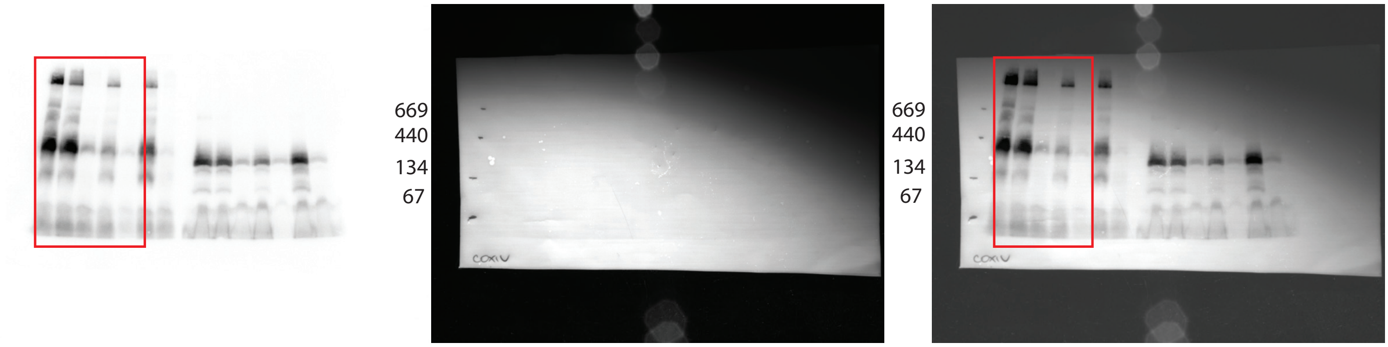

Figure 3F, ATP5A blot

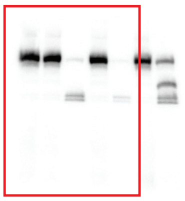

Figure 3G Source Data

Chemiluminescent signal used in figure	Colorimetric image to show molecular weight markers	Merge

Figure 3G, MRPL45 and MRPS23 blots

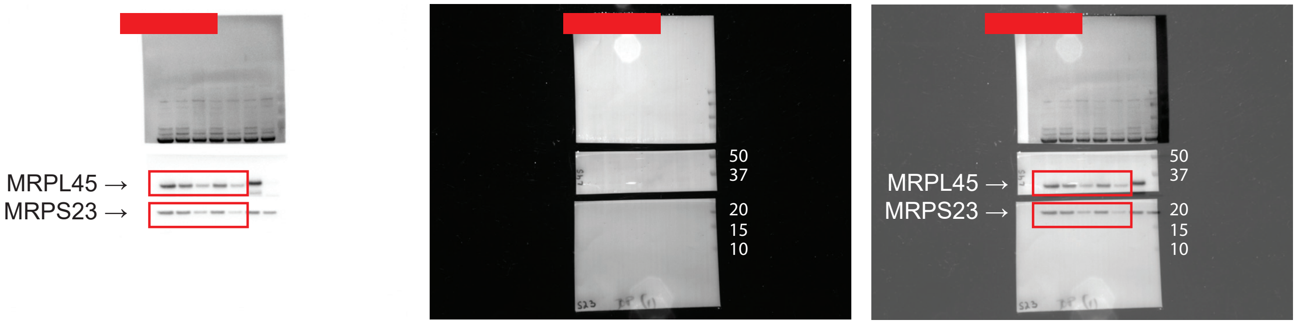

Figure 3G, FLAG blot

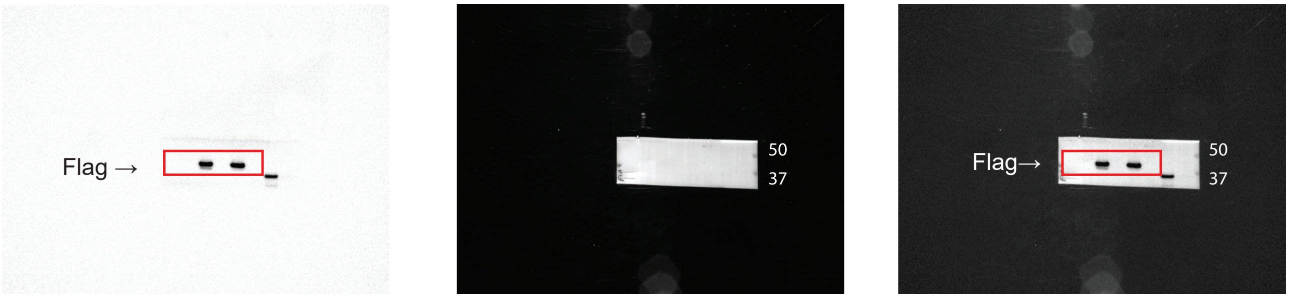

Figure 3G, MRPL44 and MRPS6 blots

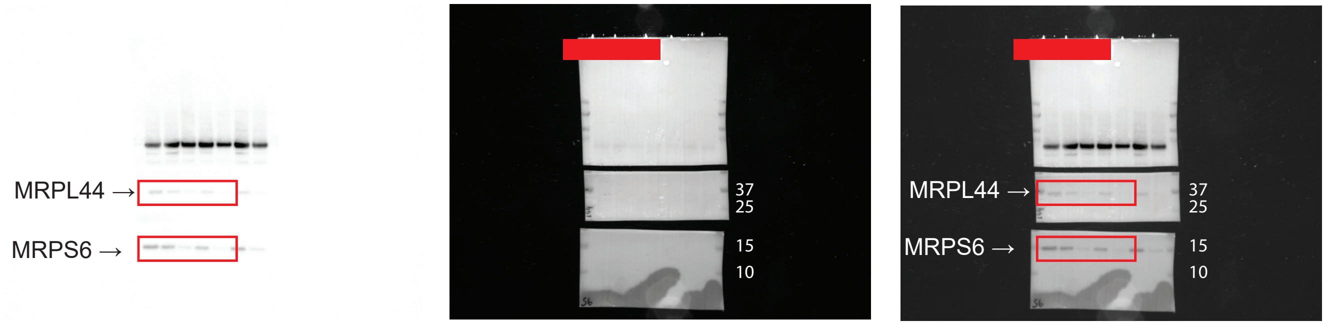

Figure 3G, MRPL49 and OXA1L blots

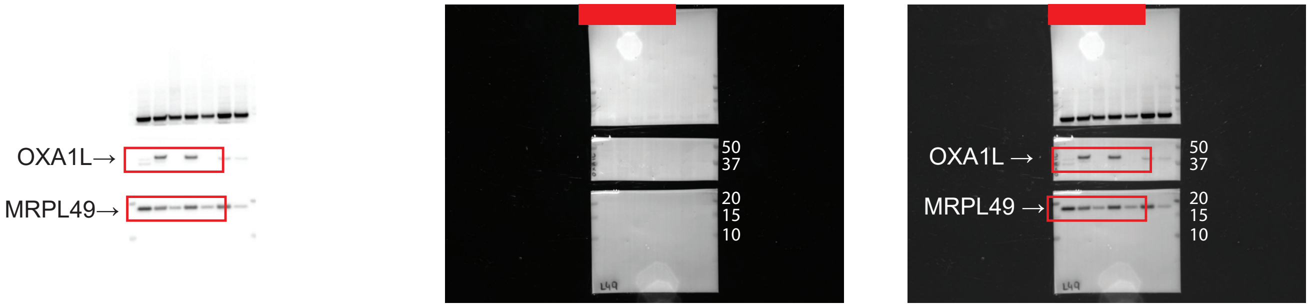

Supplement: Supplementary file 6 — Source Data for Figure 3 [file EMMM-10-e9060-s005.pdf]
