# Supplementary figures and images for: OXA1L mutations cause mitochondrial encephalopathy and a combined oxidative phosphorylation defect
Source: EMBO Mol Med. 2018 Sep 10;10(11):e9060. doi: 10.15252/emmm.201809060 (PMC6220311; doi:10.15252/emmm.201809060)

Figure 4B Source Data

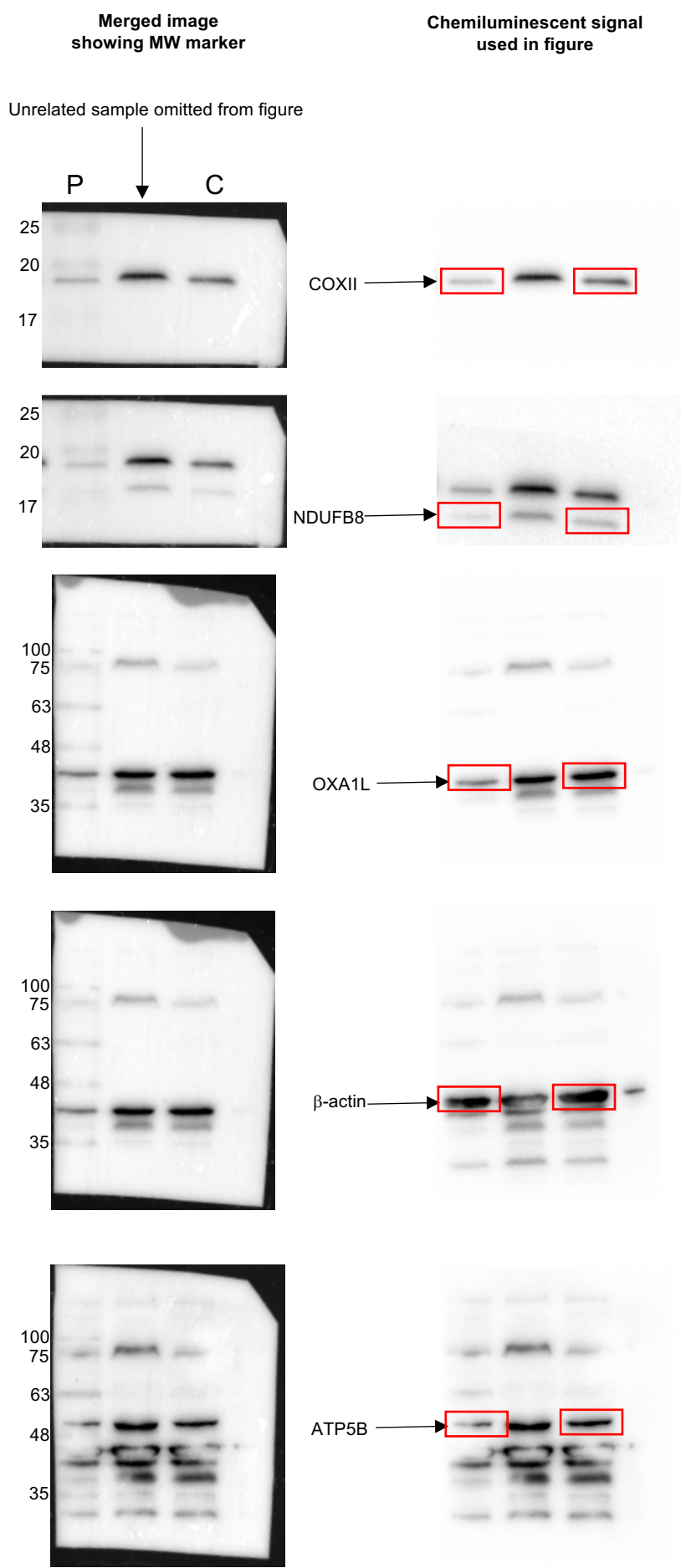

Figure 4B Source Data

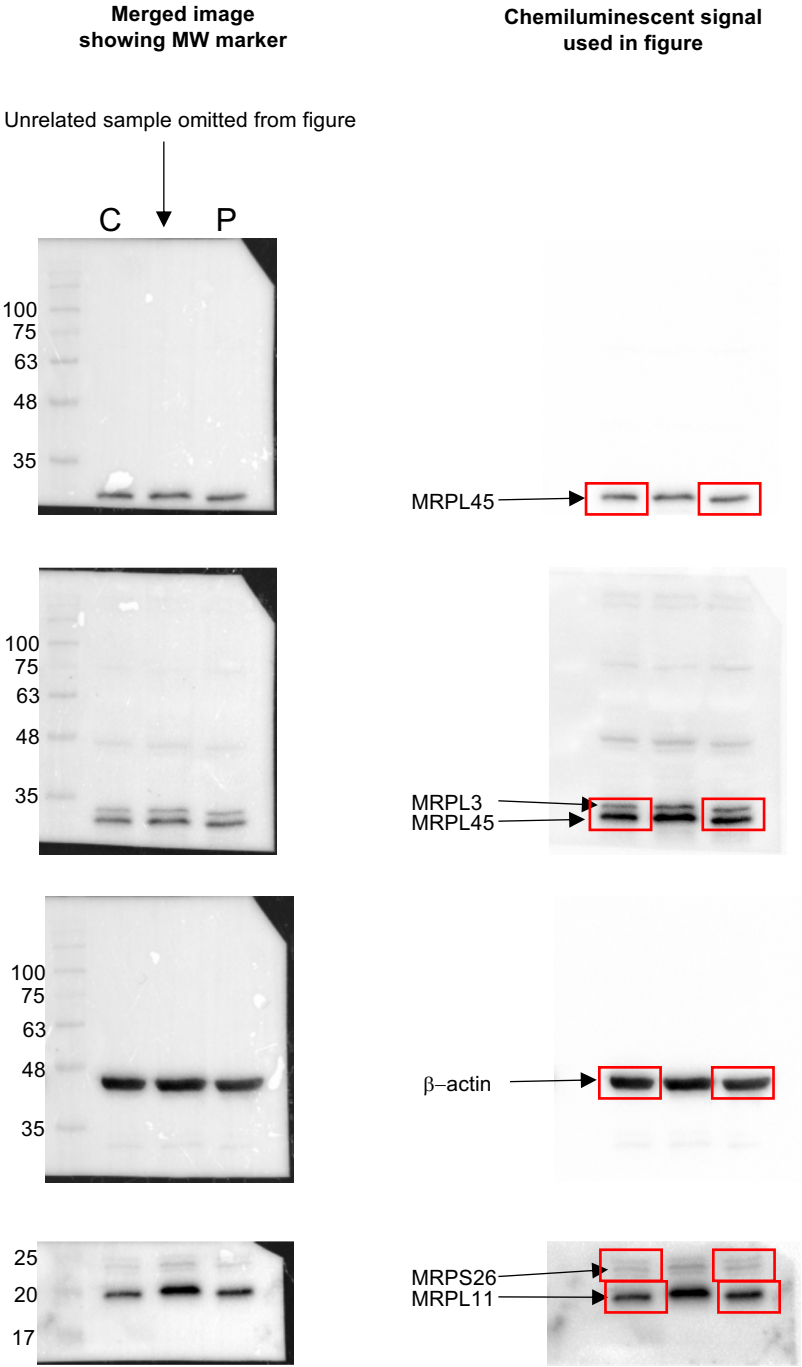

Supplement: Supplementary file 7 — Source Data for Figure 4 [file EMMM-10-e9060-s006.pdf]
